# Diseases of Children

**Published:** 1894-07-21

**Authors:** 


### Medical Progress.

### DISEASES OF CHILDREN.

**Heart Inflammation in Children.**—In the Lumleian lectures Dr. Octavius Sturges[1] points out that the early diagnosis of this affection is extremely difficult, and depends largely on the presence of pericardial rubbing. Rheumatism is, of course, the great cause of carditis, under which term the writer includes endocarditis and pericarditis. Three forms are recognised—(1) active carditis, an extremely serious and often fatal condition; (2) passive carditis, which is recognised only by the ultimate effects; and (3) chronic carditis, or pericarditis with effusion. The onset of acute carditis, which often takes place in the course of a slight attack of rheumatism, is characterised by restlessness, pallor, dyspnœa, delirium, and sometimes vomiting. Heart pain and tenderness over the cardiac region may be present. The illness often runs a rapid course to a fatal termination, due to cardiac failure. Of sixteen cases of acute carditis seen by Dr. Sturges four recovered and twelve died. In all the fatal cases the pericardium was adherent, but in only one was there extensive valvular disease. In its earliest stages this disease is to be suspected from the altered rhythm and tumultuous action of the heart, and later on by the appearance of pericardial friction and an increase in the area of cardiac dulness. Pericardial friction is the most obvious and the most important sign of this form of carditis, but careful and frequent examinations are required as it is often difficult to make out. The onset of pericarditis in a child who is apparently passing through an attack of ordinary endocarditis is not infrequent, and is of grave import. In his second lecture Dr. Sturges described a series of careful measurements made by Dr. Garrod of the normal area of superficial cardiac dulness, with a view to the diagnosis of pericarditis from an enlargement of this area. The writer then discussed the earliest recognisable signs of endocarditis in children. Amongst these he enumerated tumultuous, quickened, uneven, and reduplicated cardiac sounds, a temporary tricuspid murmur, and a marked accent commencing the first sound. The development of a mitral murmur is gradual, and it will often be absent during the course of a first attack of rheumatism. The period of quiescence as regards all acute symptoms, when the patient has passed from medical observation, is the time in which organic changes take place in the mitral valve. In the third lecture, Dr. Sturges described the effects of left-sided endocarditis on the right and uninflamed side of the heart. The impairment of muscular action in the left ventricle impedes the onward flow of blood, and thus tends to engorgement of the pulmonary vessels. The result is seen in distension of the right ventricle, which relieves itself by a tricuspid reflux, as shown by the presence of a tricuspid murmur. This murmur is, therefore, of great importance as marking the presence of endocarditis at a period before a mitral murmur has been developed. On the subject of the presystolic murmur the writer's experience is against the view that it is dependent on mitral stenosis, as this condition is seldom found post mortem. As regards prognosis in carditis, he considers it bad in that form of rheumatism in which the patient suffers from attacks repeated so frequently as almost to form one continuous illness. On the subject of treatment Dr. Sturges is chiefly critical and remarks that he has not obtained satisfactory results from the ice-bag as recommended by Dr. Lees.

**Rheumatic Endocarditis.**—In a clinical lecture, Dr. A. C. Cotton[2] concludes that from fifty to sixty per cent. of children who have had acute or subacute rheumatism are discharged from hospital with valvular disease the most frequent form of which is mitral insufficiency. The progress of endocarditis is often very slow, and is not necessarily marked by symptoms. Amongst the definite rheumatic signs now recognised are "growing pains," erythema, chorea, and pleurisy. In many cases of endocarditis in children compensation is well maintained. In others it is not, and the cardiac chambers dilate, the præcordial region is bulged forward, the rhythm is disturbed, and usually the heart's action is accelerated. The child becomes sensible of præcordial distress, wasting and anæmia are prominent signs, and recurrent attacks of broncho-pneumonia are common. In the treatment of endocarditis he employs rest, nourishing food, codliver oil, iron, arsenic, and—in uncompensated cases only—digitalis.

**Chorea.**—At the Medical Congress, Sir Dyce Duckworth[3] read a paper on *the rheumatic nature of chorea.* He maintained that chorea is not only related to rheumatism, but is a manifestation of that disease, in the same way as tonsillitis, erythema, urticaria, purpura, periosteal and integumentary nodules are recognised as manifestations of the rheumatic diathesis. He finds no difference between the endocarditis of chorea and the endocarditis of rheumatism. The site of the brain lesion in chorea he locates in the cortical motor areas, or in the basal ganglia, or in both. Definite pathological changes have not hitherto been discovered in these regions, but the writer points out that there is a marked instability and vulnerability in the great cerebral centres during childhood, which may be further intensified by the action of the rheumatic poison, without the presence of any gross lesion. The determining factor of an attack of chorea is shock or strain of the nervous system. He finds more benefit from arsenic than from any other drug. Dr. Churton[4] describes a severe case of chorea which he successfully treated with chloroform and morphine. The patient was a girl aged 17 years, who developed violent choreic movements, for the relief of which chloral in twenty-grain doses proved useless. One-sixth of a grain of morphine was then given hypodermically, and chloroform was administered for a few minutes. The dose of morphine was gradually increased to half a grain, after which the patient slept for nine hours, and had no recurrence of the very violent movements. The *eye treatment of chorea* is the subject of a paper by Dr. Ambrose L. Ranney.[5] He finds that most cases of chorea are based on a well-marked neuropathic or tubercular predisposition. About 70 per cent. of all cases of chorea suffer from hypermetropia, usually latent. Choreic subjects are usually rapidly cured by eye treatment alone. He describes several cases in which, by the use of glasses and tenotomy when required, he had procured rapid relief of all symptoms.

**Epidemic Roseola.**—An important communication has been made by Dr. Clement Dukes[6] on the distinguishing features of epidemic roseola (sometimes called "rose rash" or German measles), and the differential diagnosis between it and measles and scarlet fever. He points out that in some cases roseola will simulate the characters of measles, and in others those of scarlet fever, but holds that the three diseases are quite distinct and separate entities. Further, an attack of roseola is no protection against measles or scarlet fever, and Dr. Dukes is of opinion that the cases described as two attacks of measles or scarlet fever in rapid succession are in reality examples of unrecognised roseola in the first or second instance. A full statement of the distinguishing characteristics of roseola is given which may be summarised as follows: (1) Prevalence in spring and summer; (2) the premonitory symptoms are slight, with the exception of sore throat; (3) the incubation period is commonly eighteen days; (4) the eruption begins as minute rosy red dots on the scalp and face; (5) the lymphatic glands—notably those in the axilla, groin, and posterior cervical region—are enlarged, tender, and hard like peas; (6) there is no marked feeling of illness on the part of the patient; (7) the tongue is clean or only slightly furred, the pulse is not markedly increased in rapidity, and the temperature varies from normal to 103 deg. or 104 deg.; (8) the symptoms subside rapidly; (9) the infective period lasts from ten to fourteen days; (10) there are no sequelæ, and the termination is usually complete recovery in a fortnight. While the above are the features of a typical case, Dr. Dukes also notes that the eruption may in some cases takes the form of patches, indistinguishable from those of measles, while in others there may be a general peeling as complete as in the worst cases of scarlet fever, although in scales rather than in flakes. The treatment is summed up as follows: Five days in bed, three days indoors, six days in the fresh air, and then complete disinfection.

**Scorbutus.**—Dr. T. C. Railton[7] has described three cases of *scurvy in infants.* The patients presented well-marked signs of this affection, effusion under the periosteum being a prominent feature. Two of the cases made a rapid recovery under antiscorbutic diet, but the third died of broncho-pneumonia, a frequent complication. Another very well marked example of infantile scorbutus is recorded by Dr. Ling Taylor.[8] A female child aged eleven months, who had been fed exclusively on a certain brand of condensed milk, had suffered for four months from a series of symptoms variously ascribed to Pott's disease of the spine, *morbus coxæ,* and rheumatism. There was a distinct prominence over the first and second lumbar vertebræ, and the child was unable to sit up. The arms and hands could be moved freely without pain, but the legs were almost entirely powerless. Other signs of scurvy, namely, swollen and spongy gums, swelling around the femur and tibia with extreme tenderness to touch, and œdema of the eyelids were also present, and led Dr. Taylor to a correct diagnosis. A rapid recovery followed on the administration of Pasteurised milk and orange juice. The cause of this affection was traced directly to the use of an improper diet, and Dr. Taylor remarks that "nearly all of these infants have been nourished, or more exactly starved, on proprietary, artificial, or canned foods." In a recent communication Dr. G. A. Sutherland[9] describes two cases of hæmatoma of the dura mater associated with scurvy. In the first patient, aged two years, there was found at the necropsy a considerable amount of intracranial hæmorrhage. The surface of the brain was covered with blood clot, and the base was lined with a thick yellow membrane. Two subcortical hæmorrhages were present in the frontal region, one on each side. In the second case, an infant aged fourteen months, the whole surface of the brain was covered with a thick purplish mass which could be separated into several layers. The brain was extremely atrophied, and the cortex was in parts stained an orange yellow. From the history of these cases and the signs present the writer is led to ascribe the lesions mentioned to scorbutic disease. An interesting discussion on the subject of scurvy in children has recently taken place at the New York Academy of Medicine.[10] Reference was made to seventy-two cases which had been observed by the various speakers. Dr. Northrup defined the disease as one of malnutrition, dependent upon the long use of improper food, and the absence of fresh food or fruit juice. Dr. Forscheimer had found orange juice and fresh milk the most successful means of treatment. Dr. Lewis Smith remarked on the frequency of rickets and the rarity of scurvy amongst children, and was of opinion that the latter disease was frequently overlooked. He had never seen scurvy appear in infants who were being fed on breast milk. Dr. Jacobi met with most of his cases in cold, damp weather. He had found cases of the affection in infants of six weeks and four months. He strongly recommended phosphorus as a therapeutic agent. Amongst cases met with by Dr. Emmett Holt the following mistaken diagnoses had been made, namely, sarcoma, tubercular osteitis of the knee, and infantile paralysis. He regarded scurvy as a purely dietetic disease, rapidly cured by diet alone, and little influenced by medication of any kind. Dr. Lester Carr pointed out that the greatest swelling is near the lower end of the femur, which has the first epiphysis to appear and the last to unite. Dr. Max Einhorn thought that scurvy was due to increased alkalinity of the blood. The question of sterilised milk and proprietary foods as causes of scurvy was also under discussion. Dr. Northrup denied that the use of sterilised cow's milk would lead to scurvy, but held that the prolonged use of an insufficient food was the efficient cause.

1 B.M.J., Vol. i., 1894, pp. 505, 561, 624. 2 Chicago Clin. Rev., Jany., 1894. 3 The Lancet, April 7th, 1894, p. 891. 4 B.M.J., March 24th, 1894, p. 633. 5 N. Y. Med. Rec., May, 1894, pp. 551, 582. 6 The Lancet, Vol. i., 1894, p. 791. 7 The Lancet, March 3rd, 1894, p. 532. 8 Amer. Med. and Surg. Bulletin, Feb. 1st, 1894, p. 137. 9 Brain, No. lxv., 1894. 10 Amer. Med. and Surg. Bulletin, March 15th, 1894.

